# Supplementary material for: The Different Metabolic Responses of Resistant and Susceptible Wheats to Fusarium graminearum Inoculation
Source: Metabolites. 2022 Aug 6;12(8):727. doi: 10.3390/metabo12080727 (PMC9413380; doi:10.3390/metabo12080727)
Supplement: Supplementary file 1 [file metabolites-12-00727-s001.zip › metabolites-1826451-supplementary.pdf]

Supplementary file

The Different Metabolic Responses of Resistant and Susceptible Wheats to *Fusarium graminearum* Inoculation

Caixiang Liu [1,2,†], Fangfang Chen [3,4,†], Laixing Liu [5], Xinyu Fan [6], Huili Liu [1,2], Danyun Zeng [1,2] and Xu Zhang [1,2,7,*]

1 State Key Laboratory of Magnetic Resonance and Atomic and Molecular Physics, National Center for Magnetic Resonance in Wuhan, Wuhan Institute of Physics and Mathematics, Innovation Academy for Precision Measurement of Science and Technology, Chinese Academy of Sciences, Wuhan 430071, China; cxliu@126.com (C.L.); liuhuili@apm.ac.cn (H.L.); zengdanyun@apm.ac.cn (D.Z.)
2 University of Chinese Academy of Sciences, Beijing 100049, China
3 Songjiang Yujian High School affiliated to Shanghai Foreign Language School, Shanghai 201600, China; fangfangchen007@163.com
4 Molecular Biotechnology Laboratory of Triticeae Crops, Huazhong Agricultural University, Wuhan 430070, China
5 School of Management Wuhan Institute of Technology, Wuhan 430205, China; lxliu@126.com
6 State Key Laboratory of Component-based Chinese Medicine, Tianjin University of Traditional Chinese Medicine, Tianjin 300193, China; f17320023170@163.com
7 Wuhan National Laboratory for Optoelectronics, Huazhong University of Science and Technology, Wuhan 430074, China
* Correspondence: zhangxu@apm.ac.cn; Tel.: +86-27-87197056, Fax: +86-27-87199291
† These authors contributed equally to this work.

Table S1. Assignment of NMR data for metabolites in FHB-resistant (Sumai 3) and -susceptible (Annong 8455) wheat varieties inoculated with ddH_2O, wild type (WT), *TPS1*⁻ and *TPS2*⁻.

no.	metabolites	moieties	δ^1H (ppm)	$\delta^{13}C$ (ppm)
1	isoleucine	δ-CH_3	0.94 (t) [a]	13.7
	(Ile)	γ-CH_3	1.01 (d)	17.5
		β-CH	1.97 (m)	38.7
2	leucine	δ'- CH_3	0.96 (d)	23.5
	(Leu)	δ- CH_3	0.97 (d)	24.8
		γ-CH	1.69 (m)	26.9
		β-CH_2	1.72 (m)	42.9
3	valine	γ'- CH_3	0.99 (d)	19.8
	(Val)	γ- CH_3	1.04 (d)	20.9
		β-CH	2.27 (m)	31.7
		α- CH	3.61 (d)	63.4
4	threonine	γ- CH_3	1.33 (d)	20.8
	(Thr)	β-CH	4.24 (m)	68.6
		α- CH	3.57 (d)	63.4
		COOH		175.6
5	alanine	β- CH_3	1.48 (d)	17.9
	(Ala)	α-CH	3.78 (q)	53.4
		COOH		178.7
6	arginine	δ- CH_2	3.23 (t)	43.3
	(Arg)	γ- CH_2	1.68 (m)	26.5
		β- CH_2	1.90 (c)	29.3
		α- CH	3.77 (t)	57.3
7	methionine	γ-CH_2	2.65 (t, 7.5)	32.0
	(Met)	β-CH_2	2.17 (m)	32.5
		α-CH	3.78 (m)	56.7
		S-CH_3	2.14 (s)	16.7
8	γ-aminobutyrate	γ- CH_2	3.02 (t)	42.3
	(GABA)	β- CH_2	1.91 (qu)	26.7
		α- CH_2	2.30 (t)	37.3
		COOH		184.2
9	glutamate	γ- CH_2	2.36 (m)	34.7
	(Glu)	β- CH_2	2.02, 2.08 (m)	29.8
		α- CH	3.76 (t)	57.6
10	glutamine	γ- CH_2	2.45 (m)	32.3
	(Gln)	β- CH_2	2.14 (m)	27.5

		α- CH	3.76 (t)	55.1
		COOH		175.0
11	aspartate	β'-CH	2.82 (dd)	37.6
	(Asp)	β-CH	2.67 (dd)	43.2
		α-CH	3.90 (dd)	53.2
		COOH		175.2,178.1
12	asparagine	β'-CH	2.96 (dd)	36.1
	(Asn)	β-CH	2.86 (dd)	35.9
		α-CH	4.01 (dd)	53.2
		COOH		174.5,174.2
13	phenylalanine	C2, 6, ring	7.33 (m)	124.3
	(Phe)	C3, 5, ring	7.43 (t)	127.5
		C4, ring	7.38 (m)	127.1
14	tryptophan	C7H, ring	7.55 (d)	111.1
	(Trp)	C6H, ring	7.29 (t)	121.1
		C5H, ring	7.20 (t)	118.5
		C4H, ring	7.74 (d)	117.6
		C2H, ring	7.33(s)	126.2
		COOH		175.1
15	tyrosine	C2, 6H, ring	7.19 (d)	131.1
	(Tyr)	C3, 5H, ring	6.90 (d)	115.4
		COOH		175.3
16	histidine	5-CH	7.07 (s)	119.1
	(His)	3-CH	7.85 (s)	137.6
17	sucrose	Glc-C1H	5.41 (d)	96.2
		Glc-C2H	3.57 (dd)	72.1
		Glc-C5H	3.83 (c)	63.3
		Fru- C3H	4.22 (d)	77.3
		Fru- C4H	4.06 (t)	75.1
		Fru- C6H	3.83 (c)	63.2
18	α-glucose	C1H	5.24 (d)	95.9
		C2H	3.55 (dd)	74.3
		C4H	3.42 (dd)	72.8
19	β-glucose	C1H	4.65 (d)	98.7
		C2H	3.25 (t)	77.3
		C3H	3.49 (t)	78.9
		C4H	3.41 (dd)	72.8
		C6H	3.90 (dd)	64.3
20	raffinose	Gal-C1H	5.01 (d)	101.8
		Gal-C4H	4.01 (d)	72.6

		Gal-C5H	3.90 (m)	70.2
		Glc-C1H	5.43 (d)	95.6
		Fru-C3H	4.22 (t)	77.2
		Fru-C4H	4.06 (t)	75.3
21	fructose	5-CH	4.04, m	84.2
		4-CH	4.01 (m)	79.1
		1-CH	3.68 (d, 3.7)	65.8
22	myo-inositol	2-CH	4.07, t	75.1
		1,3-CH	3.52 (dd)	75.3
		4,6-CH	3.61 (t)	73.9
		5-CH	3.29 (t)	77.1
23	acetate	α-CH_3	1.92 (s)	26.3
24	pyruvate	CH	2.39 (s)	31.1
		C=O	\	209.9
25	succinate	α-CH_2	2.41 (s)	37.5
		COOH		184.6
26	lactate	CH_3	1.33 (d)	22.5
		CH	4.12 (q)	71.5
		COOH		185.2
27	formate	CH	8.46 (s)	169.3
28	fumarate	CH=CH	6.52 (s)	139.2
		COOH		177.5
29	citrate	α', γ' CH	2.66 (dd)	49.1
		α, γ CH	2.55 (dd)	49.1
		COOH		180.1,184.5
30	α-ketoglutarate	γ- CH_2	3.02 (t)	40.2
	(α-KG)	β- CH_2	2.44 (t)	32.1
31	malate	β'-CH	2.37 (dd)	43.7
		β-CH	2.67 (dd)	43.3
		α-CH	4.31 (dd)	73.4
32	choline	N-(CH_3)$_3$	3.20 (s)	54.5
		N-CH_2	3.52 (m)	70.2
		O-CH_2	4.07 (m)	58.5
33	phosphocholine	N-(CH_3)$_3$	3.22 (s)	56.7
	(PC)	N-CH_2	3.61 (m)	
34	glycine betaine (GB)	N-(CH_3)$_3$	3.27 (s)	54.1
35	ethanolamine (EA)	β- CH_2	3.83 (t)	56.3
36	dimethylglycine	CH_3	2.94 (s)	\
37	adenosine	C1′H, ribose	6.06 (d)	

		2-CH	8.33 (s)	
		8-CH	8.24 (s)	155.5
38	uridine	C1'H, ribose	5.90 (d)	104.5
		C6, ring	7.88 (d)	145.8
39	guanosine	8-CH	8.01 (s)	141.0
		5′-CH_2	5.90 (d)	91.0
40	hypoxanthine	2-CH	8.20 (s)	\
		7-CH	8.22 (s)	\
41	inosine	12-CH	8.32 (s)	
		2-CH	6.10 (d)	
42	deoxy adenosine monophosphate	8-CH	8.27 (s)	\
	(dAMP)	C1H of ribose	6.14 (d)	89.9
43	p- hydorxy cinnamic acid	7-CH	7.55 (d)	132.1
	(p-HCA)	5-CH	6.97 (d)	118.1
44	chlorogenic acid	8-CH	6.39	124.3
		2, 4-CH	6.93 (d, 8.3)	118.5
		3, 5-CH	7.35 (d, 8.3)	143.2
		6-CH	4.25 (d, 2.90)	73.6
45	thymidine	6-CH	7.65 (s)	140.0
		5′-CH_2	6.30 (t)	87.0

[a] Multiplicity: s, singlet; d, doublet; t, triplet; dd, doublet of doublets; qu, quintet; m, multiplet. \,The signals were not determined.

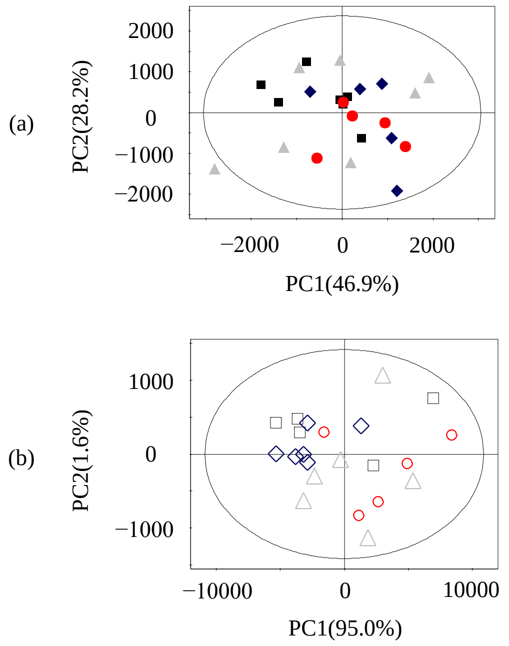

Figure S1. PCA scores plots of the NMR data for FHB-resistant Sumai 3 (a) and -susceptible wheat Annong 8455 (b) respectively. The numbers in parentheses indicate the overall variance explained in the first two principal components. Grey (triangle), black (square), red (circle), and blue (diamond) indicate metabolites in both FHB- resistant (solid symbol) and -susceptible wheat (open symbol) inoculated with ddH_2O, WT, *TPS1*$^-$ and *TPS2*$^-$ respectively.
